# The curious case of an internal pilot in a multicentre randomised trial—time for a rethink?

**DOI:** 10.1186/s40814-016-0113-8

**Published:** 2016-12-13

**Authors:** Jonathan Alistair Cook, David John Beard, Johanna Rosemary Cook, Graeme Stewart MacLennan

**Affiliations:** 1 Centre for Statistics in Medicine, Nuffield Department of Orthopaedics, Rheumatology and Musculoskeletal Sciences, Botnar Research Centre, Nuffield Orthopaedic Centre, University of Oxford, Windmill Road, Oxford, OX3 7LD UK; 2 Surgical Intervention Trials Unit, Nuffield Department of Orthopaedics, Rheumatology and Musculoskeletal Sciences, Botnar Research Centre, Nuffield Orthopaedic Centre, University of Oxford, Windmill Road, Oxford, OX3 7LD UK; 3 Nuffield Department of Primary Care Health Sciences, University of Oxford, Radcliffe Observatory Quarter, Woodstock Road, Oxford, OX2 6GG UK; 4 Health Services Research Unit, University of Aberdeen, Health Sciences Building, Foresterhill, Aberdeen, AB25 2ZD UK

**Keywords:** Randomised trial, Pilot study, Recruitment, Monitoring, Multicentre, Feasibility

## Abstract

Multicentre randomised trials are complex projects with many operational uncertainties. The embedding of a formal check upon study progress and viability at a pre-specified time point (sometimes referred to as an ‘internal pilot’) is becoming increasingly common within multicentre pragmatic randomised trials. However, it is worth considering this practice. We argue that most, if not all, multicentre trials have reassessment of the recruitment strategy and study processes whilst the study is running. Additionally, we propose discontinuation of the ‘internal/external pilot study’ terminology. Instead, we suggest for an alternative taxonomy along with greater recognition of the process of refinement which routinely occurs in trials and transparent reporting of it.

## Main text

Multicentre randomised trials are complex projects with many operational uncertainties particularly related to recruitment of participants, set-up of sites, timing and practicalities of randomisation, and data collection. Whilst, very valuable, no amount of preliminary (feasibility, piloting) work can fully remove or anticipate all problems. Given this, a check upon progress and review of problems when the study is at an early stage has much to commend it. The embedding of a formal check upon study viability at a pre-specified time point (sometimes referred to as an ‘internal pilot study’) is becoming increasingly common within large multicentre pragmatic randomised trials [1–5]. Based upon a review of the website of one research programme, around a half of trials funded in 2015 had an ‘internal pilot’ [2]. *‘*Internal pilots’ can have a somewhat ethereal nature to their existence, and they may not be explicitly reported in published trial report [6]; though with the publication of trial protocols, they are becoming more readily identified [7].

Funders naturally want to ensure funds are used efficiently and may request a formalised assessment of recruitment early in a trial’s lifecycle to identify problems and potentially curtail trials that are failing [2, 5]. However, it is worth considering this practice. On a simple level, the increasingly common labelling of this as an ‘internal pilot study’ is, in our view, unhelpful. A pilot in the natural sense of the word *has* to be external to the thing to which it is a pilot for. In practice, the use (or not) of the pilot data in the analysis of the trial appears to be the main dividing line between an ‘internal’ and ‘external’ pilot study [5]. This terminology appears to have originated from the desire to include a formal check of key assumptions underlying the sample size calculation [3, 4, 8]. At the more substantive level, it is important to recognise that most, if not all, multicentre trials undergo some modification, and also have regular reassessment of the recruitment strategy, during their life course. Typically mo﻿difications relate to the recruitment strategy and the data collection process though other changes, including adjusting the sample size or replacing the primary outcome, are not that uncommon [3–5]. Perhaps some industry-funded drug trials are the exception where identical study processes can be adopted from a previous study, and the only distinguishing feature is the active compound. There is though, a sense that all trials are unique in the specific combination of methodology, location, timing and personnel. All trials have their own ‘story’. This is particularly true of pragmatic or comparative effectiveness trials, which tend to be intentional ‘one-offs’. Many, if not all, of the features of an internal pilot will go on in most (if not all) multicentre trials with only the formal assessment at a specified time the distinguishing feature. Is there ever a trial conducted without consideration of the recruitment progress against expectations, or a review of the data collection process? Indeed, this is one of the responsibilities of trial management groups, and similarly, funders may undertake periodic checks on progress. We note that the timing of a one-off assessment and the specification of stop-go recruitment criteria (typically of the form of *X* centres having recruiting at least one participant and/or a total of *Y* participants recruited by month *Z*) are difficult to get right. The desire for explicit and applicable criteria needs to be balanced against the multifaceted nature of the decision to progress or not. Relevant considerations include factors external to the trial (e.g. availability of wider resources to support a study with serious recruitment problems). To recognise this is not to deny the value of a planned assessment, more the recognition that it is difficult to anticipate and to formalise all the eventualities and considerations which go into making decisions relating to what is a complex, and in some regards, unique project. Furthermore, regular monitoring of the study process is a fundamental part of good trial science. It is not without reason that trial co-ordination and management are increasingly being recognised for its necessity, expertise and value, and as an emerging area for research [9]. Reports of trial results however tend to downplay this perhaps due to fears of how the study might be viewed.

Where can we go from here? First, our proposal is to stop using the prefixes ‘internal’ and ‘external’; true pilot studies are always external. A trial which incorporates a single-planned check of a key aspect of the study (such as recruitment) could instead refer to a ‘stop-go’ assessment or phase. In Table 1, we suggest an alternative taxonomy of approaches for developing and modifying randomised trial design with particular reference to the recruitment strategy. Clarification of when to use each approach and what the main output is also given. We note that the routine monitoring and stop-go assessment approaches are not mutually exclusive options, and that the latter is unlikely to be carried out without some form of the former. Second, trial protocols and reports should be more transparent about the potential and actual modification of trial processes and important design features (e.g. changes to outcomes), and whether it was a part of a pre-planned refinement process or a response (very likely a legitimate one) to an unforeseen event. Due to the difficulties of a one-off assessment, the ‘routine monitoring’ approach may be the most appropriate within-trial approach, and in our view better reflects what often occurs even when a stop-go assessment is specified. The ACCEPT guidance offers a helpful structure to think through where uncertainties lie when deciding upon which approaches to adopt [10]. Third, there is an underdeveloped area of refinement of methodology and processes during the life course of a trial which needs exploration and development. *BMC Pilot and feasibility studies* is the natural home for publishing the findings of such work.

**Table 1:** Approaches to developing and modifying the trial design with particular reference to the recruitment strategy

Approach	Conception	When to use	Key purpose	Main output
Feasibility study	Separate study	Substantial uncertainty about whether it is possible or how to implement aspects of the trial design and conduct (e.g. delivery of the interventions and recruitment process)	Exploration of the feasibility of a trial	Assessment of the feasibility of a trial
Pilot trial	Separate study	Limited refinement of the trial design, processes and recruitment strategy anticipated	Refinement of trial design and processes prior to commencement of the main trial	Minor modifications to trial design and confirmation of practicality
Stop-go assessment	Early in the recruitment period of the main trial	Substantial adjustment of recruitment strategy and study processes is considered likely	One-off within study assessment of recruitment and study processes to allow adjustment of the strategy (including the possibility of early cessation of recruitment)	Single reassessment of the recruitment and data collection strategy
Routine monitoring	Throughout the recruitment period of the main trial	Minor modifications to study processes and recruitment strategy anticipated	Regular monitoring to allow periodic within study modification	Periodic updating of the recruitment strategy and study processes

## Conclusion

Refinement of study processes and reassessment of the recruitment strategy are key elements of most, if not all, multicentre randomised trials. There is a sense in which most have features of an ‘internal pilot study’. We recommend discontinuation of the terminology of ‘internal’ and ‘external’ pilot studies as it is in our view unhelpful. There should be greater recognition of the process of refinement which occurs routinely in trials, increased clarity about any planned formal assessment, and more transparent reporting of this. We have suggested an alternative way to think about this along with a proposed taxonomy. Further research in this area is needed.

## Abbreviations

ACCEPT: Acceptance checklist for clinical effectiveness pilot trials
